# Transcriptome Landscape at Different Developmental Stages of a Drought Tolerant Cultivar of Flax (*Linum usitatissimum*)

**DOI:** 10.3389/fchem.2017.00082

**Published:** 2017-11-09

**Authors:** Prasanta K. Dash, Rhitu Rai, Ajay K. Mahato, Kishor Gaikwad, Nagendra K. Singh

**Affiliations:** National Research Centre on Plant Biotechnology, Pusa Institute, New Delhi, India

**Keywords:** flax/linseed, RNAseq/transcriptome, drought stress, *de novo* assembly, Illumina MiSeq

## Introduction

Drought is a global phenomenon that affects productivity of all field crops. Comparatively, flax is prone to drought stress and concomitant yield penalty. Owing to vagaries of climate change, erratic monsoon and global warming; drought research received significant attention in model as well as field crops. While, priority crops like rice, wheat, corn, and canola witnessed significant advances in drought research (Aprile et al., 2009; Hayano-Kanashiro et al., 2009; Lenka et al., 2011; Zhang et al., 2014), limited impetus has been accomplished in an industrially important crop flax/linseed (*Linum usitatissimum*; Gupta and Dash, 2015). Recently, transcriptome data for legumes such as pigeonpea (*Cajanus cajan*) and its wild relative *C scaraboides* have been available (Nigam et al., 2017) for translational research. Flax, a dual purpose crop grown for fiber and seed oil, entered genomics research with decoding of its genome in 2012 (Wang et al., 2012). Since then genomic resources in flax are accumulating (Dash et al., 2014, 2015; Gupta et al., 2017; Shivaraj et al., 2017) to accelerate its varietal improvement program. Of late, development of high-throughput RNA sequencing revolutionized analysis of eukaryotic transcriptomes (Wang et al., 2009) and facilitated elucidating pathways and mapping of novel genes. Globally, meager genomic information are available in flax for translational research with information only on related *Linum* species like *L. bienne, L. grandiflorum*, and *L. leonii* (Johnson et al., 2012), In a recent study, fiber development in flax was elucidated using the RNA-Seq information generated in *L. usitatissumum* (Zhang and Deyholos, 2016). Thus, there is an urgent need among *Linum* research community to saturate genomic information on drought, salt, cold, and heat-stress mechanisms operative in flax genotypes grown across the globe. In this endeavor, transcriptome analysis of a moderately drought tolerant flax cultivar T-397 was accomplished to delineate biochemical pathway and genes operative in imparting tolerance to drought in this cultivar of Indian flax.

To accomplish translational research for drought tolerance in flax, no genomic data are available from any drought tolerant cultivar. Also, information related to gene expression profiling in flax is limited. This is the first report of high resolution transcriptome data from a moderately drought tolerant flax cultivar of an Indian origin. This dataset will be a cardinal genomic resource in annotating and understanding the genes and the intrinsic pathways involved in drought tolerance in flax. The data will further help to identify transcripts that are detectable under normal growing conditions encompassing different stages of flax growth as well as identifying genes involved in warding off drought at vegetative as well as reproductive stages in flax. It can also be used for gene discovery and/or comparative transcriptome analysis with the other related species. Besides being a useful resource for delineating the molecular basis of drought tolerance in flax, the inherent information can be used by plant breeders in flax breeding strategies.

## Experimental design, materials, and methods

### Plant materials

Seeds of linseed variety T-397 were obtained from project coordinating unit (Linseed), Kanpur, India. Seeds were sown in 25 cm diameter plastic pots filled with mixture of peat soil, vermiculite, and river sand in 1:1:1 ratio. The seed-laden pots were kept in dark for 3 days. After germination, the pots were shifted to tissue culture room having 12 h photoperiod and 24°C/18°C (day/night) temperature. Shoot tissues were collected from 20 d old five independent plants with three biological replications while bud and inflorescence tissues were collected in a similar manner after onset of flowering (60 d). Same tissue types from 15 independent plants were pooled to represent a homogenous sample. All the tissues were frozen in liquid nitrogen before storing at −80°C.

### Total RNA extraction, quality check, and sequencing

Total RNA was isolated using 500 mg of shoot, flower, and bud separately using commercially available RNA isolation kit (Qiagen). Extracted RNA was treated with TURBO DNA-free™ kit to get rid of chromosomal DNA contamination. Quality of the isolated RNA was checked by 1% denaturing agarose gel electrophoresis and quantified by using a Nanodrop. Equal amount of total RNA having A_260/280_ ratio >2.0 were supplied to service provider for library preparation. Illumina TruSeq RNA sample preparation kit was used to prepare the cDNA librarry. Four micro-gram of total RNA was used to isolate polyA mRNA using oligodT coupled magnetic beads. Subsequently, the mRNA from three different samples were pooled, fragmented and cDNA was synthesized using random primers and reverse transcriptase (Super-Script II). The double stranded cDNA after an end repair process (Klenow fragment, T4 polynucleotide kinase and T4 polymerase), was ligated to Illumina paired end (PE) adaptors. The Library was enriched using 15 cycles of PCR, purified and diluted to a final concentration of 4 nM and run at a concentration of 9 pM on MiSeq Instrument (Illumina, USA) using MiSeq Reagent Kit v2 (300 Cycles) with 2 × 150 PE sequencing.

### Preprocessing of raw reads

Raw reads were filtered with Q20 quality trimming (removal of low quality reads with average quality score <20 and trimming of low quality bases from the end of reads). The quality filtering was performed to remove adapter sequences with sequence pre-processing Trimmomatic v0.30 (Bolger et al., 2014) software with following parameters: sliding window length of 20, leading and trailing threshold quality value of 20. After trimming, reads with read length less than 40 bp were discarded.

### *De novo* transcriptome assembly

High quality Illumina raw reads with phred score ≥25 were used for assembly. The *de novo* assembly of these processed reads was accomplished using Trinity assembler (version r2014-07-17) (Grabherr et al., 2011) with the following parameters: Jellyfish Memory of 300G, minimum contig length of 1,000 base pair and heap-space of 7 (Marçais and Kingsford, 2011). Subsequently, the assembly statistics were obtained using custom perl script (Bradnam et al., 2013). The assembled transcripts were further clustered into non-redundant unigene set using CD-HIT-EST software with default parameters (% similarity >95%). The quality of assembled unigenes transcripts was evaluated by mapping the total high quality reads to final unigene set number of reads to assembled transcripts using Bowtie2 (Li and Durbin, 2009).

## Results

The transcriptome assembly has turned out to be a method of choice for the discovery and characterization of novel transcripts involved in diverse pathways in eukaryotes (Wang et al., 2009; Surget-Groba and Montoya-Burgos, 2010). Availability of next generation sequencing technology has expedited identification and characterization of genes based on transcriptome approach in several crops. As a first step toward identifying drought specific genes, RNA-seq library in flax was prepared from the pooled samples of shoot, bud, and inflorescence. A total of 47,004,561 paired end reads were obtained after quality filtering. The cleand reads were obtained by filtering raw reads by removing adapters, low-quality, and ambiguous reads. Pre-assembly statistics of raw reads along with distribution of contigs and contigs length is presented in Table 1. The mapping statistics of high quality filtered data obtained are presented in Table 2. The GC content of the assembled data was 44.94% while 55.06% was AT content.

**Table 1:** Pre-assembly statistics of raw reads along with bar chart representing the distribution of assembled contigs with number of contigs in the X-axis and Range of contig length in the Y-axis.

**Sample ID**	**Total number of reads**	**Total number of bases**	**Mean read length (bp)**
Pool_R1.fastq	47,004,561	6,757,288,060 (6.7 GB)	143.75
Pool_R2.fastq	47,004,561	6,611,966,755 (6.6 GB)	140.67
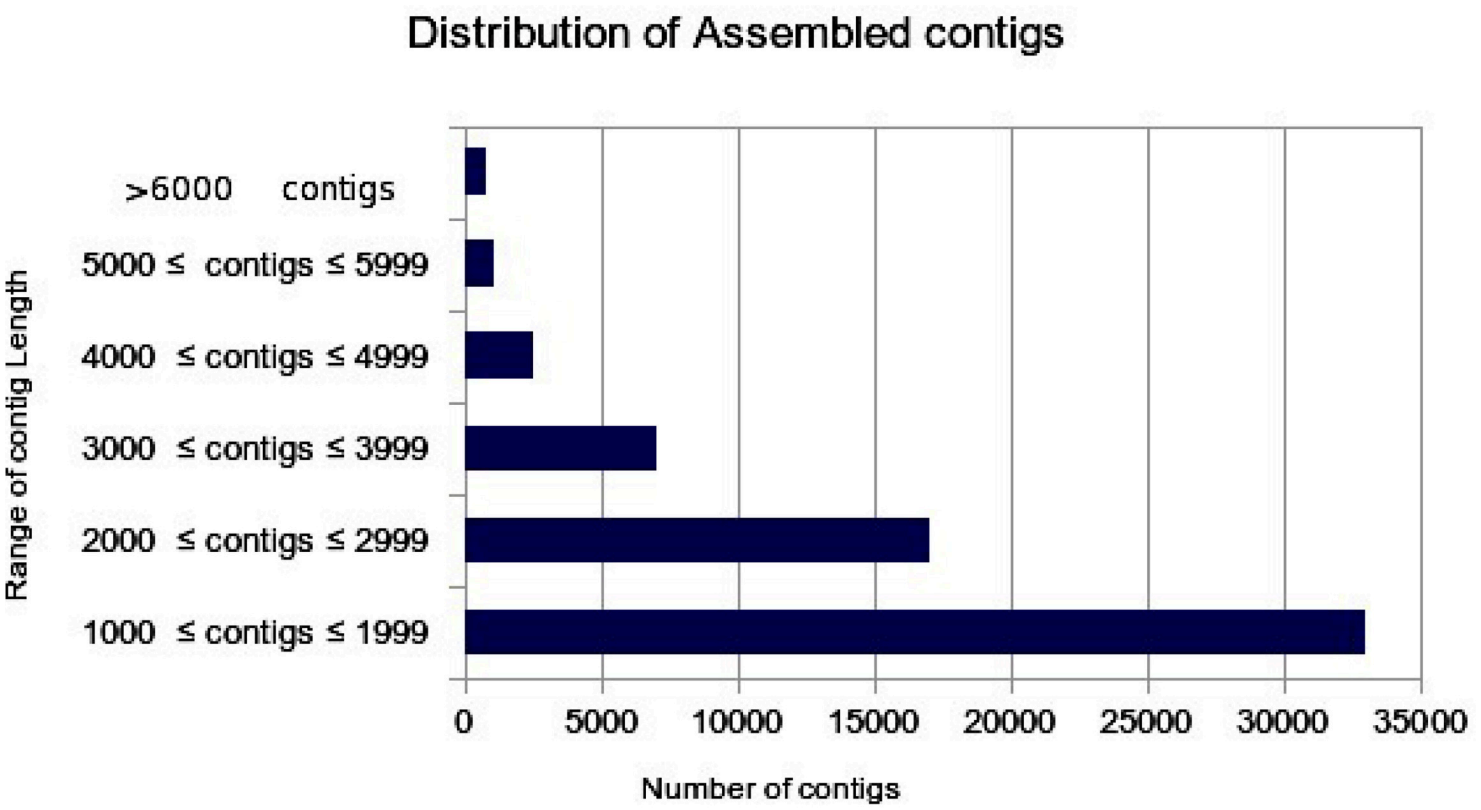			

**Table 2:** Statistics of *de novo* assembly and UniGenes along with mapping statistics of *L. usitatissimum* transcriptome.

**Assembly metrics**	**Trinity assembly statistics**	**UNIGENE statistics**
Number of contigs	61,563	39,330
Total size of contigs	137,099,459 bp	83,672,257 bp
Longest contig size	15,047 bp	15,047 bp
Mean contigs size	2,227 bp	2,127 bp
Median contigs size	1,921 bp	1,836 bp
N50	2,422 bp	2,283 bp
contigs %A	27.50%	27.50%
contigs %C	22.27%	22.27%
contigs %G	22.67%	22.67%
contigs %T	27.56%	27.56%
contigs %GC	44.94%	44.94%
**PROPERLY PAIRED END READS MAPPING TO TRINITY ASSEMBLED CONTIGS**		
Total Number of PE Reads	4,70,04,561	
Unique Mapping PE Reads	2,21,53,868	
Multi Mapping PE Reads	1,62,43,478	
Un-Mappable PE Reads	86,07,215	
Discordant Mapping PE Reads	7,02,265	
Percentage of Mappable PE Reads	88.11%	

The filtered data assembled using Trinity software (Grabherr et al., 2011) resulted in 61,563 transcript contigs with N_50_ value of 2.4 kb, with average length of 1.9 kb. The transcripts were further analyzed using Cluster Database at High Identity with -EST i.e. CD-HIT_EST software (Nakasugi et al., 2013). A total of 61,563 transcripts were clustered into 39,330 UniGene's using CD-HIT-EST (Li and Godzik, 2006; Nakasugi et al., 2013). The clustered UniGene's has average length of 1.8 kb with N_50_ value of 2.2 kb and. Subsequently, 52,678 CDS were identified using transdecoder from 39,330 UniGene's. The statistics of transcriptome assembly and UniGene prediction are represented in Table 2. Assessment of transcriptome assembly was accomplished using unigenes formed after clustering. Subsequently, Bowtie2 was used to align the HQ reads on unigenes and the mapping statistics are represented in Table 2. Our result revealed that out of 47,004,561 PE reads 88.11% are mapable pairs reflecting the high quality of the *de novo* generated assembly. While, 22,153,568 are unique mapping reads; 16,243,478 reads mapped to multiple sites and 8,607,215 reads were un-mapable. The discordant/unmapable reads most likely correspond to low expressed transcripts/unsatisfactory coverage or are of aberrant nature.

### Link to deposited data and information to the user

Transcriptome profile of *L. usitatissimum* was generated from the polyA-enriched cDNA library prepared by pooling equal amount of total RNA extracted from shoot, flower and buds separately. The short reads were filtered, processed, assembled, and analyzed as described in the previous section. The complete data from the current study was submitted at NCBI under the BioProject ID PRJNA338739. The raw data for this project were deposited at SRA database at NCBI with the accession number SRR4034646 (http://www.ncbi.nlm.nih.gov/sra/SRR4034646). Users can download and reuse the data for research purpose with an acknowledgment and by quoting this paper as reference to the data.

## Conclusion

This is the first report of a transcriptome dataset of an Indian flax cultivar T-397. We used next-generation RNA sequencing of leaf, shoot, bud, and flowering inflorescence to identify the genes involved in intrinsic drought tolerance in this cultivar. The data can be further used for identifying SSR loci and markers to be used in flax improvement program specific to drought tolerance.

## Author contributions

PD and RR conceived the study, performed the experiments, analyzed the data with help from AM, KG, and NS. PD wrote the manuscript with input from all authors. All authors read and approved the manuscript.

### Conflict of interest statement

The authors declare that the research was conducted in the absence of any commercial or financial relationships that could be construed as a potential conflict of interest.
